# Exploring post-Covid-19 condition in children and young people 3.5 years after infection: a mixed-methods analysis from the CLoCk study

**DOI:** 10.1186/s12889-026-27520-z

**Published:** 2026-05-19

**Authors:** Emma Dhir-Hewitt, Fiona Newlands, Roz Shafran, Terence Stephenson, Alvin Richards-Belle, Emma Dalrymple, Trudie Chalder, Tamsin Ford, Lana Fox-Smith, Isobel Heyman, Shamez N. Ladhani, Malcolm G Semple, Terry Y. Segal, Olivia Swann, Elizabeth Whittaker, CloCk Consortium, Snehal M Pinto Pereira

**Affiliations:** 1 Division of Surgery & Interventional Science, Faculty of Medical Sciences, University College London, London, WC1E 6BT UK; 2 UCL Great Ormond Street Institute of Child Health, 30 Guilford Street, LondonLondon, WC1N 1EH UK; 3 Division of Psychiatry, University College London, London, UK; 4 Department of Psychological Medicine, Institute of Psychiatry, Psychology and Neuroscience, King’s College London, De’Crespigny Park, London, SE5 8AF UK; 5 Department of Psychiatry, University of Cambridge, Hershel Smith Building Cambridge Biomedical Campus, Cambridge, CB2 0SZ UK; 6 Immunisations and Vaccine Preventable Diseases, UK Health Security Agency, 61 Colindale Avenue, London, NW9 5EQ UK; 7 Centre for Paediatric and Neonatal Infection, St. George’s University of London, Cranmer Terrace, London, SW17 0RE UK; 8 NIHR Health Protection Research Unit for Emerging and Zoonotic Infections, Institute of Infection, Veterinary and Ecological Sciences, University of Liverpool, Liverpool, UK; 9 Liverpool Institute of Child Health and Wellbeing, Alder Hey Children’s Hospital NHS Foundation Trust, Eaton Road, Liverpool, UK; 10 Department of Paediatrics and Adolescence, University College London Hospital, London, UK; 11 Centre for Medical Informatics, Usher Institute, University of Edinburgh, Edinburgh, EH16 4TL UK; 12 Department of Paediatric Infectious Diseases, Imperial College Healthcare NHS Trust, London, W2 1NY UK

**Keywords:** Post-COVID services, Post-COVID-19 Condition, Children and young people, Paediatric, Long COVID, SARS-CoV-2, Mixed methods

## Abstract

**Background:**

Comprehensive data on the persistence of Post-COVID-19 Condition (PCC; also known as Long COVID) and its impact on children and young people (CYP), incorporating their own perspective, is crucial to enhance our understanding of the condition, improve service provision and inform clinical management.

**Methods:**

We examine long-term symptoms, health, and well-being among CYP persistently meeting PCC criteria up to 3.5-years after SARS-CoV-2 infection (when they were aged between 11-to-17-years), using a mixed-methods approach. 68 CYP from the CLoCk study who persistently met PCC criteria at 3- (April-June 2021), 6- (July–September 2021), 12- (January-March 2022), and 24-months (January-March 2023) post-infection were invited to complete an additional follow-up (October–November 2024). The survey assessed current symptoms and health status using validated measures, and symptom experiences through open-text responses. Quantitative data were analysed descriptively; qualitative data were analysed using thematic framework analysis. Findings were integrated using a convergent parallel design.

**Results:**

50 CYP completed the survey; of these 42 (84%) responders continued to meet the PCC definition 3.5-years post-infection. All 42 (100%) reported tiredness and 34 (81%) reported 5 + symptoms 3.5-years post-infection. Qualitative analysis reinforced tiredness as a central symptom, alongside co-occurring symptoms that impact daily life. While quantitative and qualitative findings largely converged, context as to why CYP reported high levels of impact were only available from qualitative data.

**Conclusions:**

CYP with PCC persisting for 3.5-years post-infection experience multiple symptoms of wide-ranging severity and disruption to daily life, education and social participation.

**Supplementary Information:**

The online version contains supplementary material available at 10.1186/s12889-026-27520-z.

## Introduction

Post-COVID-19 Condition (PCC), also known as Long COVID, refers to symptoms that persist for at least two months following a COVID-19 infection and cannot be explained by an alternative diagnosis [1]. While most children and young people (CYP) recover, we found a minority experience symptoms up to 2-years post-infection [2]. PCC symptoms are diverse, but include fatigue, difficulty concentrating, and respiratory issues [3]. These symptoms can adversely affect CYP’s well-being, daily functioning, educational participation, peer relationships and engagement in leisure activities [4]. This is particularly important because adolescence represents a critical developmental window characterised by rapid biological, cognitive, and social change. Disruptions during this period may have lasting consequences for educational attainment, identity formation, social integration, and mental health. Persistent health difficulties during this period may therefore extend beyond immediate symptom burden, potentially influencing trajectories into adulthood, including employment prospects, social participation, and long-term healthcare needs. Comprehensive longitudinal data incorporating CYP’s own perspectives are essential to understand the persistence and impact of PCC and to inform clinical management and service provision.

PCC prevalence in CYP is wide ranging, from 1-to-70**%** [5–7]. This variability may be attributed to several factors, including differences in study design (e.g., timing of PCC ascertainment), population sampled, and PCC definitions employed [7]. Many studies fail to use established PCC criteria, focusing on single specific symptoms rather than aligning with WHO or other established definitions [8]. Importantly, most studies have relatively short follow-up periods [8], limiting understanding of PCC persistence over time.

The Children and Young People with Long COVID (CLoCk) study, is a prospective cohort of over 31,000 CYP in England aged 11-to-17-years at the time of PCR-testing in 2020–21 [9]. The study previously demonstrated that PCC prevalence declines over time; at 3-months post-infection, 25% of test-positive CYP met the PCC definition, but only 7% continued to meet this definition persistently at 3-, 6-, 12-, and 24-months post-infection [2]. Among CYP with persistent PCC from 3-to-24-months (N = 68), two symptom clusters were observed. While many reported tiredness, a subgroup presented with a multi-symptom profile, including tiredness, shortness of breath, and additional symptoms, whereas others predominantly experienced fatigue only [10]. Despite heterogeneous symptom profiles, even those experiencing few symptoms found their symptoms to be debilitating with an impact on their mental health and well-being [10]. These findings highlight the importance of continued follow-up to fully understand the long-term impact of PCC.

With few exceptions [11], there is limited follow-up of paediatric PCC beyond 2-years. Ideally, longitudinal studies would track all SARS-CoV-2-infected CYP over time to map symptom trajectories and long-term outcomes. In practice, however, resource and logistical constraints often necessitate more focused approaches. In this study, we invited the 68 CYP from the CLoCk study who consistently met the PCC definition at 3-, 6-, 12-, and 24-months post-infection, to participate in a further follow-up approximately 3.5-years post-infection. By combining quantitative and qualitative data on symptoms, health and well-being, we aim to offer novel insights into the longer-term outcomes of a well-characterised group with persistent PCC. We acknowledge that, in the absence of a contemporaneous control group at this stage of follow-up, causal inference regarding symptom attribution is limited; rather, our objective was to describe longer-term experiences and impacts among CYP with previously documented persistent PCC.

## Methods

CLoCk is a cohort study of 31,012 CYP living in England aged 11-to-17-years when they PCR-tested for SARS-CoV-2 (between 09/2020–03/2021); the number of data collection sweeps varied depending on the CYP's month of PCR-testing [12]. Here, our target population was the sub-sample of CYP who PCR-tested positive for SARS-CoV-2 in January-March 2021 and persistently met the PCC definition (see below) at 3- (April-June 2021), 6- (July–September 2021), 12- (January-March 2022) and 24-months (January-March 2023) post-testing [2, 13] (N = 68). These CYP were sent an additional questionnaire at approximately 3.5-years post-infection (October–November 2024) which forms the basis of the current manuscript. The study was approved by Yorkshire and the Humber–South Yorkshire Research Ethics Committee (reference:21/YH/0060); all participants provided informed consent.

At inception, the UK Health Security Agency provided data on sex at birth, age and region of residence at time of PCR-testing, and the 2019 English Index of Multiple Deprivation (IMD; a proxy for socioeconomic status [14]). CYP reported their ethnicity at enrolment.

## 3.5-year Questionnaire

The 3.5-year questionnaire was based on previous questionnaires [12]. We asked about 21 symptoms currently being experienced (broadly aligning with the International Severe Acute Respiratory and Emerging Infection Consortium Paediatric COVID-19 questionnaire [15]); validated health scales (Strengths and Difficulties Questionnaire (SDQ) [16], Short Warwick Edinburgh Mental Wellbeing Scale (SWEMWS) [17], Chalder Fatigue Scale [18], and, a measure of quality of life/function, the EQ-5D-Y [19]) were also included. Free-text questions included a request to describe *‘how severe your symptoms have been and how they have affected your daily life’*, and, space to *' tell us about Long COVID and how it has affected you’* (see supplementary section for copy of questionnaire).

### PCC 3.5-years post-infection

The Delphi research definition of PCC in CYP [20] was operationalised at the time of questionnaire completion (i.e., ~ 3.5-years post-infection) as experiencing ≥ 1 symptom from the pre-specified symptom list (described above) AND problems with at least one of: mobility OR self-care OR doing usual activities OR having pain/discomfort OR feeling very worried/sad, based on the EQ-5D-Y scale. CYP meeting this operationalised research definition ~ 3.5-years post-infection were classified as having PCC at that time.

### Quantitative analysis

First, we compared questionnaire responders to non-responders. Second, we described symptom profiles of responders in totality and stratified by PCC status at 3.5-years by tabulating the total number of symptoms reported, prevalence of individual symptoms, self-rated health, symptom severity, and impact. We also report the prevalence of those who talked to their doctor about PCC and stayed overnight in hospital for PCC. We examined 3.5-years post-infection health and well-being status (via validated scales) stratified by PCC at 3.5-years. Specifically, we compared responders with persistent PCC from 3-months to 3.5-years post-infection to those whose PCC resolved by 3.5-years (i.e., present from 3-to-24-months but not at 3.5-years). We used Fisher’s exact, Mann–Whitney or t-tests, as appropriate.

Missing data was minimal because almost all numerical questions examined were compulsory. Data management and analysis were performed using STATAv18.0. Quantitative data from CLoCk are available via the UK Data Service (ID: 9203) [21].

### Qualitative analysis

Considering only responders continuing to meet the definition of PCC 3.5-years post-infection, qualitative data were analysed by a single rater (FN), using Thematic Framework Analysis. Focusing on open-ended responses to the two questions described above, this involved a multi-staged structured process: familiarisation, framework development, coding, framework application, charting data into a matrix, and data interpretation [22]. FN developed the framework (after discussion with co-author SMPP) using a combination of symptoms identified by participants in the open-text responses and the 21 symptoms described above. Data were indexed by systematically applying the framework matrix of symptoms to each open-text response. Indexed data were charted into rows representing symptom categories (e.g., fatigue/tiredness, breathlessness, loss of taste/smell), with columns capturing impact on daily/overall life. Patterns and relationships across symptom categories were summarised, enabling theme identification. The initial identified codes and emerging themes were revised iteratively following discussions with coauthors (EDH, SMPP, RS and ARB), ensuring a comprehensive representation of participants’ experience. These analyses informed an overarching narrative describing the lived experiences of participants with PCC up to 3.5-years post-infection. NVivo was used to support analysis; the framework matrix was developed and charted in Excel.

### Data integration

**Fig. 1:**
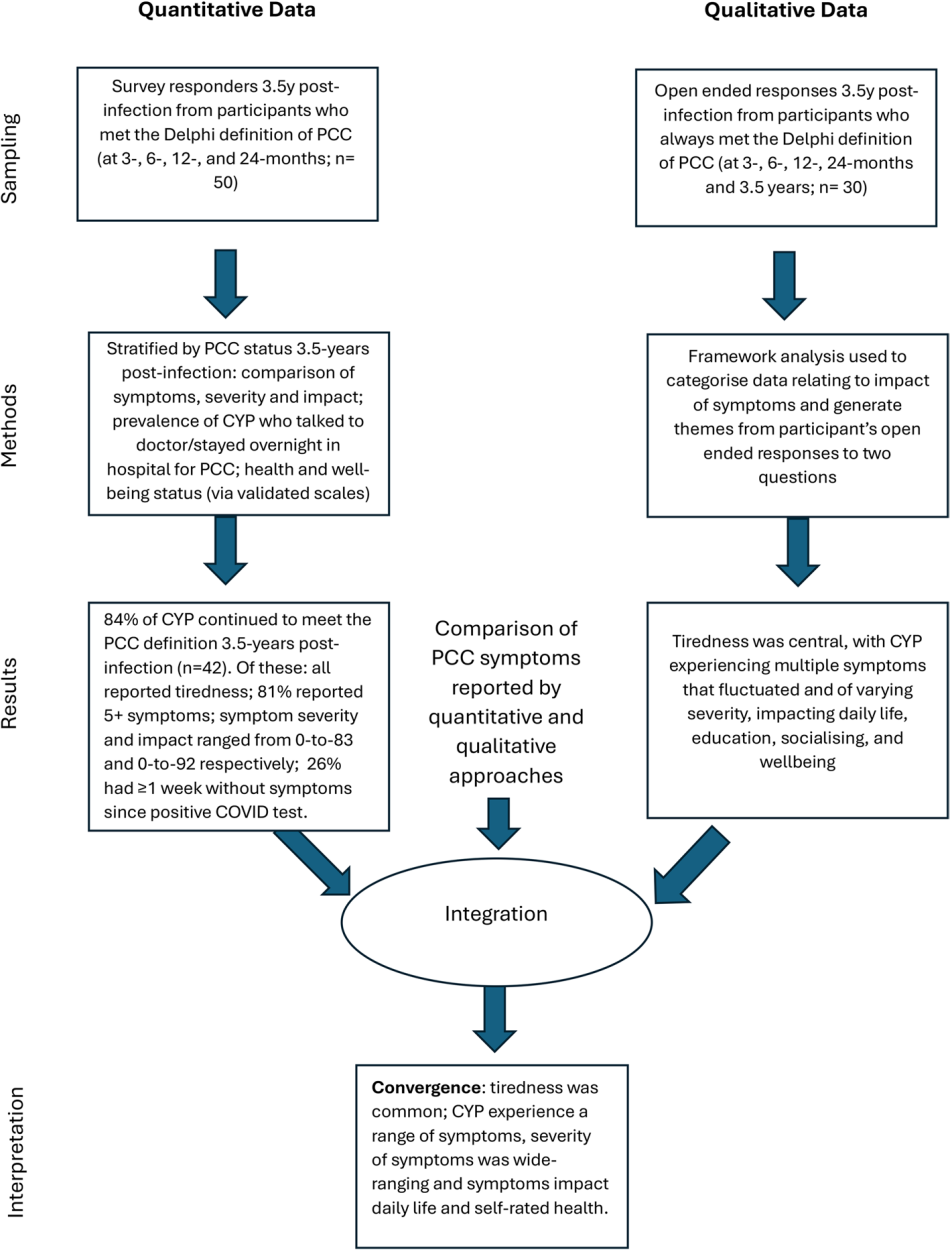
Application of the parallel mixed methods design

We compare quantitative findings to qualitative descriptions to identify potential areas of similarities and differences in CYP’s experiences of persistent PCC. We applied a convergent parallel mixed methods design [23, 24] (see Fig. 1). This approach is suitable because our quantitative and qualitative data were collected and examined concurrently. It also allows for quantitative and qualitative findings to carry equal weight and data integration to occur via comparison, thereby determining similarities or differences across the two data sources.

## Results

Of 68 invited, 50 (73.5%) CYP responded (Table 1; Supplementary Fig. 1). Compared to non-responders (n = 18), responders (n = 50) were somewhat more likely to be female than male (86% vs. 72%) and aged 15-to-17-years rather than 11-to-14-years at their index-infection (76% vs. 61%). Responders were more likely to reside in deprived areas, with 46% in the most deprived IMD quintiles (1–2) compared to 17% of non-responders. While there were some regional differences between responders and non-responders, they were broadly similar in terms of ethnicity.

**Table 1:** Demographics (N(%)) of the target population*, non-responders and responders included in the quantitative and qualitative analysis

	Target population* (*N* = 68)	Non-responders (*N* = 18)	Responders included in:	
			quantitative analysis (*N* = 50)	qualitative analysis (*N* = 30)
Prevalence		26.5%	73.5%	44.1%
Sex				
Female	56 (82.4)	13 (72.1)	43 (86.0)	26 (86.7)
Male	12 (17.7)	5 (27.8)	7 (14.0)	4 (13.3)
Age at index test/infection (years)				
11–14	19 (27.9)	7 (38.9)	12 (24.0)	9 (30.0)
15–17	49 (72.1)	11 (61.1)	38 (76.0)	21 (70.0)
Ethnicity				
White	55 (80.9)	14 (77.8)	41 (82.0)	24 (80.0)
Asian/Asian British	7 (10.3)	2 (11.1)	5 (10.0)	3 (10.0)
Mixed	4 (5.9)	1 (5.6)	3 (6.0)	2 (6.7)
Black/African/Caribbean	1 (1.5)	1 (5.6)	0	0
Other	1 (1.5)	0	1 (2.0)	1 (3.3)
Region of residence				
East Midlands	4 (5.9)	2 (11.1)	2 (4.0)	1 (3.3)
East of England	10 (14.7)	1 (5.6)	9 (18.0)	8 (26.7)
London	12 (17.7)	4 (22.2)	8 (16.0)	4 (13.3)
North East England	1 (1.5)	0	1 (2.0)	1 (3.3)
North West England	6 (8.8)	0	6 (12.0)	5 (16.7)
South East England	13 (19.1)	3 (16.7)	10 (20.0)	5 (16.7)
South West England	12 (17.7)	6 (33.3)	6 (12.0)	3 (10.0)
West Midlands	8 (11.8)	2 (11.11)	6 (12.0)	2 (6.7)
Yorkshire and the Humber	2 (2.9)	0	2 (2.0)	1 (3.3)
IMD quintile**				
1 (most deprived)	10 (14.7)	1 (5.6)	9 (18.0)	4 (13.3)
2	16 (23.5)	2 (11.1)	14 (28.0)	9 (30.0)
3	18 (26.5)	7 (38.9)	11 (22.0)	6 (20.0)
4	12 (17.7)	5 (27.8)	7 (14.0)	7 (23.3)
5 (least deprived)	12 (17.7)	3 (16.7)	9 (18.0)	4 (13.3)

^*^The target population was the sub-sample of CYP who PCR-tested positive for SARS-CoV-2 in January, February or March 2021 and persistently met the PCC definition at 3-, 6-, 12- and 24-months post-infection; see methods for details

^**^IMD: 2019 English Index of Multiple Deprivation. The Index of Multiple Deprivation (IMD) was calculated from the CYP’s small local area level based geographic hierarchy (lower super output area) at the time of study enrolment and used as a proxy for socio-economic status. We report IMD quintiles from most (quintile 1) to least (quintile 5) deprived

Median time since index-infection and questionnaire completion was 3.7-years (Range: 3.5-to-3.9-years). Of the 50 responders, 42 (84%) continued to meet the PCC definition 3.5-years post-infection while the remaining 8 (16%) met the PCC definition from 3-to-24-months post-infection but no longer meet it 3.5-years post-infection. Although numbers are small, there were differences in symptom profiles of those continuing to meet vs no longer meeting the PCC definition 3.5-years post-infection (Table 2). For example, 3.5-years post-infection, 34/42 (81%) of those continuing to meet the PCC definition reported 5 + symptoms, compared to 1/8 (12.5%) of those no longer meeting the PCC definition (p < 0.01). Specifically, those continuing to meet the PCC definition were more likely to report chills, persistent coughs (42.9% vs 0% p = 0.04 for both), tiredness (100% vs 75%; p = 0.02) and sleeping difficulties (81% vs 37.5%; p = 0.02). Some symptoms were reported exclusively by those continuing to meet the PCC definition and with moderate-to-high prevalence (i.e. ≥ 20%); these symptoms included: loss of smell/taste, unusual chest pain, abdominal and muscle pains and earache. Those continuing to meet the PCC definition were also more likely to have spoken to their doctor about their PCC symptoms (23/42 vs 1/8; p = 0.02), score their self-rated health lower (median: 55.5 vs 78.5; p = 0.03) and report a higher symptom impact (median: 50 vs 0; p < 0.01) than those no longer meeting the PCC definition. In contrast, 3.5-years post-infection, there was no evidence of differences in mental health, well-being or fatigue between those continuing to meet vs no longer meeting the PCC definition (Table 3; p ≥ 0.29). For example, mean well-being difference was 0.3 (95% CI: −2.80, 3.50) as measured on the SWEMWS scale; Supplementary Table 1.

**Table 2:** Reported symptoms N(%), self-rated health^a^, symptom severity^a^ and impact^a^ for the quantitative analytical sample and stratified by PCC status 3.5-years post-infection

	Quantitative analytical sample N = 50	Meeting PCC definition 3.5-years post-infection *N* = 42	Not meeting PCC definition 3.5-years post-infection *N *= 8	*P* value^b^
*Prevalence*		84%	16%	
Number of reported symptoms				
0	1 (2.0)	0 (0.0)	1 (12.5)	< 0.01
1	1 (2.0)	0 (0.0)	1 (12.5)	
2	5 (10.0)	3 (7.1)	2 (25.0)	
3	4 (8.0)	2 (4.8)	2 (25.0)	
4	4 (8.0)	3 (7.1)	1 (12.5)	
≥ 5	35 (70.0)	34 (81.0)	1 (12.5)	
Specific symptoms				
Fever	3 (6.0)	3 (7.1)	0 (0.0)	1.00
Chills	18 (36.0)	18 (42.9)	0 (0.0)	0.04
Persistent cough	18 (36.0)	18 (42.9)	0 (0.0)	0.04
Tiredness	48 (96.0)	42 (100.0)	6 (75.0)	0.02
Shortness of breath	34 (68.0)	30 (71.4)	4 (50.0)	0.25
Loss of smell/taste	9 (18.0)	9 (21.4)	0 (0.0)	0.32
Unusually hoarse voice	2 (4.0)	2 (4.8)	0 (0.0)	1.00
Unusual chest pain	15 (30.0)	15 (35.7)	0 (0.0)	0.09
Unusual abdominal pain	11 (22.0)	11 (26.2)	0 (0.0)	0.17
Diarrhoea	7 (14.0)	7 (16.7)	0 (0.0)	0.58
Headaches	29 (58.0)	26 (61.9)	3 (37.5)	0.26
Unusual eye-soreness	11 (22.0)	10 (23.8)	1 (12.5)	0.67
Skipping meals	15 (30.0)	14 (33.3)	1 (12.5)	0.41
Dizziness or light-headedness	21 (42.0)	20 (47.6)	1 (12.5)	0.12
Sore throat	7 (14.0)	5 (11.9)	2 (25.0)	0.31
Unusual strong muscle pains	9 (18.0)	9 (21.4)	0 (0.0)	0.32
Earache or ringing in ears	13 (26.0)	13 (31.0)	0 (0.0)	0.09
Raised welts on skin or swelling	0 (0.0)	0 (0.0)	0 (0.0)	-
Red/purple sores/blisters on feet	0 (0.0)	0 (0.0)	0 (0.0)	-
Sleeping difficulties	37 (74.0)	34 (81.0)	3 (37.5)	0.02
Other	5 (10.0)	5 (11.9)	0 (0.0)	0.58
Did you/your parent talk to a doctor about your PCC symptoms?^c^	24 (51.1)	23 (59.0)	1 (12.5)	0.02
Did you stay overnight in hospital for PCC?^c^	1 (2.1)	1 (2.5)	0 (0.0)	1.00
Self-rated health^a^	59 (43,70)	55.5 (42,69)	78.5 (60,87)	0.03
Symptom severity^a^	35 (25,53)	39.5 (26,60)	20 (4.50)	0.08
Symptom impact^a^	50 (20,60)	50 (25,68)	0 (0,40)	< 0.01
No symptoms ≥ 1 week since positive covid-19 test^d^	15 (34.1)	11 (29.7)	4 (57.1)	0.21

*NB*: Symptom “Confusion, disorientation or drowsiness” not included in questionnaire

^a^Reported as median(IQR), scored on a scale of 0 (worst) to 100 (best) for self-rated health; 0 (not severe at all) to 100 (extremely severe) for symptom severity; 0 (no impact) to 100 (extreme impact) for symptom impact

^b^*p* value from Fisher’s exact test of association between PCC status 3.5-years post-infection and number of symptoms, specific symptoms, speaking to doctor and staying overnight in hospital; from Mann–Whitney tests between PCC status 3.5-years post-infection and self-rated health, symptom severity and symptom impact

^c^ ‘yes always’ and ‘yes some of the time’ combined together; N = 47 (talk to doctor)/48 (overnight hospital stay)

^d^ "No symptoms ≥ 1 week since positive COVID-19 test" had 6 "not applicable" responses: denominators for the 3 columns are therefore: 44, 37 and 7 respectively

**Table 3:** Validated scales 3.5-years post index-infection for the quantitative analytical sample and stratified by PCC status 3.5-years post-infection (Mean (SD))

	Quantitative analytical sample *n* = 50	Meeting PCC definition 3.5-years post-infection *n* = 42	Not meeting PCC definition 3.5-years post-infection *n* = 8	*P* value^a^
Strengths and Difficulties Questionnaire (SDQ)				
Total Difficulties	19.9 (5.8)	19.7 (5.6)	20.8 (6.9)	0.64
Emotional symptoms	6.8 (2.3)	6.7 (2.4)	7.6 (1.4)	0.29
Conduct problems	2.3 (1.7)	2.4 (1.8)	1.9 (1.6)	0.41
Hyperactivity/inattention	6.8 (2.3)	6.8 (2.2)	6.6 (2.8)	0.86
Peer relationship problem	3.9 (2.1)	3.8 (1.9)	4.6 (2.6)	0.30
Short Warwick-Edinburgh Mental Wellbeing Scale (SWEMWBS)	18.0 (4.0)	18.1 (4.1)	17.7 (3.7)	0.83
Chalder fatigue scale (CFS)				
Proportion meeting case-ness N (%)	46 (92.0)	39 (92.8)	7 (87.5)	0.51

A higher SDQ score indicates more problems; a higher SWEMWBS score indicates better mental well-being; Case-ness was defined as CFS total score ≥ 4 using bimodal scoring; Number of symptoms and the EQ-5D-Y scale at 3.5-years post-infection not shown in this table as they are part of the definition of PCC

^a^*p*-values from t-tests comparing those meeting (vs not meeting) the PCC definition at 3.5-years post-infection

Of the 50 responders, 3 (6%) were awaiting PCC treatment; all 3 of these CYP meet the PCC definition from 3-months to 3.5-years post-infection. Similarly, only 1 (2%) person was receiving PCC treatment, and they also were from the group that continued to meet the PCC definition 3.5-years post-infection. 20 (40%) CYP were receiving treatment for other conditions with a further 9 (18%) awaiting treatment. The majority of these CYP (18 and 8 respectively) belonged to the group that continued to meet the PCC definition at 3.5y post-infection. Among CYP who continued to meet the PCC definition (n = 42), perceptions of recovery varied widely. For example, 11 (26%) CYP reported having at least a week without symptoms since their positive Covid-19 test. Over a third of these CYP (15; 36%) rated their recovery below 5 on a scale from 0 (not recovered) to 10 (fully recovered), indicating limited perceived recovery. In contrast, among CYP who no longer met the PCC definition (n = 8), none rated their recovery below 5.

### Qualitative results

Out of the 42 CYP who continued to meet the PCC definition 3.5-years post-infection, 30 (44%) provided responses to at least one of the two open-text questions. The majority of these CYP were female (87%) and of White ethnicity (80%) (Table 1).

Four themes were identified, with narrative description and exemplar quotes provided below.

#### Theme 1: tiredness: a central symptom

Tiredness was commonly reported including persistent fatigue that disrupted daily functioning, requiring considerable effort to complete even basic tasks:

*Tiredness is my main problem; somedays I just want to lay down […] and not do anything* (P29).

For many, tiredness was constant, leading to challenges staying awake and focused at school and limited ability to socialise and spend time with family:

*It impacts my […] education as I am more tired than the rest.* (P21)

*I […] have significant absence from college resulting in an attendance plan. I will have to sleep after attending college and then I don't get to be sociable with family and friends.* (P9)

For some, feelings of tiredness were severe enough that they needed to rest during the day:

*I have been so tired since having Covid as well as dizzy […] I have started taking afternoon naps to help* (P11)

#### Theme 2: co-occurring symptoms

Participants described experiencing multiple symptoms simultaneously. Symptoms such as brain fog, breathlessness, dizziness, and chest tightness were frequently mentioned. Many participants reported these made daily activities, such as concentrating or engaging in physical tasks more difficult:

*Can't remember words/meanings etc. […]. So exhausted sometimes from loss of sleep.* (P17)

The combined presence of physical symptoms with cognitive difficulties added further complexity to participants’ experiences. These symptoms often disrupted routine activities, including attending school/work:

*The shortness of breath can be a bit tricky […] fatigue makes it hard for me to stay awake or even get to work.* (P12)

Participants highlighted the combined burden of multiple symptoms. For some, this meant a persistent alteration to their routines and ongoing struggles:

*I have been hospitalised for gastric dysfunction, been housebound with dystonic paralysis, always struggling with lethargy* (P20)

For some, symptoms occurred *‘randomly’* (P19) or unpredictably, as participants described days when their condition fluctuated or when certain symptoms appeared without warning:

*The chest tightness is sometimes ok, then on other days bad enough where I struggle to breathe normally.* (P21)

Lasting effects of respiratory symptoms were particularly notable; participants mentioned how breathlessness and sensory disruptions affected their lives:

*My breathing has been affected since I first had COVID, and my sense of smell and taste haven’t returned back to normal still.* (P11)

## Theme 3: symptom severity

Symptom severity varied widely. At the milder end, participants reported relatively manageable symptoms, describing them akin to coryza or noting they were able to do everything they wanted:

*Symptoms are mild and sometimes difficult to focus but relatively manageable.* (P8)

For others, symptoms were severe and altered their ability to function; in one instance resulting in hospitalisation:

*I was in horrendous pain and had to be hospitalised.* (P28)

## Theme 4: impact on daily life

Participants described the broader impact of PCC on their health, noting how persistent symptoms affected their overall wellbeing and ability to engage in daily activities:

*I seem to get ill more often […] which affects my general health and my going to work.* (P22)

Others reflected on the need to consider whether an activity was worth undertaking, given the potential consequences of worsening symptoms thereby limiting their ability to participate in activities they previously enjoyed:

*I can’t do as much as my peers or be as active as I wish without it affecting both my mental and physical health.* (P24)

Increased feelings of anxiety and distress were described including experiencing ‘*a lot of anxiety and panic about random things*’ (P17). Another described the toll on their overall health, reflecting ‘*the failures across my body’s systems and its impact on my life has been very distressing*.’ (P20). The inability to taste was particularly upsetting with one describing it as “*depressing*” (P22) they could no longer enjoy food:

*I wish I could enjoy food again […] I barely like anything anymore* (P29)

Participants expressed frustration regarding lack of clarity on the causes of their symptoms and how to manage them:

*Wish I had answers for why things are happening and some vague course of treatment. I have been left with loose prognosis and no plan for treatment because nobody can find answers* (P20)

The absence of a clear recovery path appeared to be associated with hopelessness with one participant commenting that ‘*it feels so depressing there is no recovery’* (P23).

Participants also described feelings of anxiety about their health if re-exposed to the virus. This was particularly concerning considering the lack of mandatory public health measures:

*I worry and am anxious about how COVID would affect my body if I were to get it again... [people] don't wear masks on public transport […]. Act like it doesn't exist […]. That's just a scary feeling* (P24)

The lack of protective measures left participants feeling unsupported and anxious.

### Data integration

Table 4 and Fig. 1 summarise the four identified themes by the quantitative and qualitative findings, highlighting areas of convergence and divergence.

**Table 4:** Themes identified from quantitative and qualitative findings on CYP persistently meeting the PCC definition up to 3.5-years post-infection

Theme	Quantitative (*N* = 42)	Qualitative (*N* = 30)	Quantitative and qualitative findings converge or diverge?
Tiredness as core symptom	100% of CYP continuing to meet the PCC definition 3.5y post-infection reported tiredness; almost all CYP (39/42) also met the ‘caseness’ criteria on the Chalder fatigue scale	Tiredness/fatigue was the most referenced symptom, with participants describing an overwhelming impact on education, social life and daily activities ‘*Tiredness is my main problem somedays I just want to lay down all day and not do anything’*	Convergence: tiredness is a common and central symptom
Co-occurring symptoms	34 (81%) CYP continuing to meet the PCC definition 3.5y post-infection reported 5 + symptoms 11 (26%) CYP experienced at least a week without symptoms since a positive Covid test	CYP described multiple symptoms occurring simultaneously, making daily life difficult *“Fatigue and brain fog impacts my daily life as I struggle to focus on anything.”* Symptoms occurred ‘randomly’, fluctuated or appeared without warning: *‘Chest tightness is sometimes ok, then on other days bad enough where I struggle to breathe normally.’*	Convergence: CYP continuing to meet the PCC definition 3.5y post-infection experience a range of symptoms that can fluctuate
Symptom severity	Among CYP continuing to meet the PCC definition 3.5y post-infection, severity was wide-ranging from 0-to-83 (on a scale from 0 (not severe) to 100 (extremely severe)’; median = 39.5). 23 (59%) CYP talked (or their parent talked) to a doctor about their PCC symptoms	Wide ranging symptom severity: some CYP described symptoms as mild and manageable, while others reported severe disability *“Feels like a cold, but I can still do everything I want.”* vs *“I was in horrendous pain and had to be hospitalised.”*	Convergence: CYP continuing to meet the PCC definition 3.5y post-infection experience variability in symptom severity
Impact on daily life	CYP continuing to meet the PCC definition 3.5y post-infection reported low self-rated health, high symptom impact and poor mental health/well-being, e.g., symptom impact ranged from 0-to-92 (on a scale from 0 (not severe) to 100 (extremely severe)’; median = 50)	CYP described distress, including anxiety about symptoms, frustration with lack of answers, and fears of reinfection *“It feels so depressing that there is no recovery.”* Many CYP felt unsupported due to lack of public health measures Some participants described the broader impact of PCC on their health: *‘I can’t do as much as my peers or be as active as I wish without it affecting both my mental and physical health.’*	Convergence: Symptoms impact daily life and self-rated health, with findings suggesting **high levels of anxiety, distress, and frustration among CYP** continuing to meet the PCC definition 3.5y post-infection Only qualitative analysis was able to demonstrate that many CYP felt unsupported due to lack of public health measures

Quantitative and qualitative findings mostly converged to demonstrate that (i) tiredness is a common and central symptom of PCC, (ii) CYP with PCC persistently up to 3.5-years post-infection experience a range of symptoms that can fluctuate, (iii) there is a wide-range of symptom severity and (iv) symptoms impact daily life and self-rated health. Only qualitative analysis provided context as to why CYP reported high levels of impact, pointing to frustration with lack of answers and public health measures and, reinfection fears.

## Discussion

We invited 68 CYP from the CLoCk study who persistently met the PCC definition over a two-year period to an additional follow-up 3.5-years post-infection. By examining their symptoms, health, well-being and free-text responses, we provide novel insights into the long-term impact of persistent PCC in CYP. Among 50 respondents, the majority (n = 42; 84%) continued to meet the PCC definition, with most of these CYP having a high symptom burden and healthcare engagement. For example, 81% (n = 34/42) reported experiencing 5 + symptoms 3.5-years post-infection and 59% (n = 23/42) reported speaking to a doctor about their PCC. Qualitative analysis reinforced the prominence of tiredness as a central symptom, alongside co-occurring symptoms and impact on daily life.

We acknowledge study limitations, including our sample size (N = 50 (quantitative); N = 30 (qualitative)). Ideally, we would follow-up all CLoCk CYP, but funding constraints necessitated a targeted approach. We therefore focused on following-up CYP who persistently met the PCC definition for more than 2-years post-infection. Accordingly, findings should be interpreted as exploratory, as we aimed to characterise longer-term experiences and impacts among CYP with previously documented persistent PCC rather than to draw definitive conclusions. Future data linkage efforts will allow for longer-term follow-up of the entire cohort, including those who recovered earlier, providing insights into heterogenous recovery trajectories and enhancing understanding of factors associated with recovery versus persistence. Here we were able to explore quantitively and qualitatively experiences of long-term persistence. Responders (vs non-responders) were more likely female, older, and residing in more deprived areas; this should be considered when assessing the generalisability of our findings. Assuming the extremes ─ that all 18 non-responders either still meet or no longer meet the PCC definition ─ the estimated proportion of CYP with persistent PCC at 2-years post-infection still affected at 3.5-years would range from 62%-to-88%. Thus, while persistent PCC may be relatively rare in the general population [2], our findings highlight that among those affected for 2 years, it can have debilitating longer-lasting impacts. Results may also be specific to England and not transferable to other countries with different healthcare systems/COVID-19 policies. Unlike previous CLoCk questionnaires, “confusion, disorientation or drowsiness” was not included in the symptom list. Due to cessation of community PCR-testing, we were unable to account for reinfections and therefore cannot determine whether symptoms reported over time were attributable to the index infection or to subsequent infection(s). Moreover, while our methodology allows us to quantify PCC at specific time-points, it does not provide insight into PCC persistence between data collection sweeps. Nonetheless, qualitative and quantitative findings demonstrated that some CYP experienced periods without symptoms. We emphasise our aim is to describe and not attribute cause. While symptom prevalence was high in CYP continuing to meet the PCC definition 3.5-years post-infection, many symptoms commonly reported in paediatric PCC are prevalent (albeit to a lower level) in general paediatric populations [25]. For example, pre-pandemic median prevalence of headache was 30% [25] compared with a prevalence of 62% here, among CYP that continued to meet the PCC definition 3.5-years post-infection. Despite limitations, a key strength and novelty of our methodology is integrating quantitative and qualitative data to understand PPC. Additionally, by describing symptoms and PCC impact 3.5-years post-infection, we add to sparse literature [11] regarding understanding of long-term implications of SARS-CoV-2 infection during a critical life-stage characterised by biological, cognitive, and social change. Our findings therefore contribute novel longitudinal insight into the potential enduring effects of infection at a time when developmental trajectories may be especially sensitive to disruption.

Our qualitative findings align with those previously reported from CLoCk and elsewhere [26–28], identifying fatigue as a central, debilitating symptom. Persistent tiredness was found to affect daily functioning, limit participation in school and social activities, and contribute to distress/frustration linked to unpredictability of symptoms and absence of clear treatment pathways. Importantly, the prominence of fatigue in CYP after SARS-CoV-2 infection, highlighted here and more broadly, underscores its significance. During adolescence, energy levels, cognitive stamina, and physical capacity are intertwined with developmental, educational, and social milestones. Thus, enduring fatigue can derail key developmental processes, disrupt learning trajectories, and impair psychosocial adjustment. This makes persistent fatigue in CYP not only a symptom of PCC but a potential long-term threat to health, wellbeing, and life chances. We found that some CYP self-reported recovery from COVID-19, highlighting variability in symptom profiles and illness trajectories. This is consistent with previous CLoCk findings and broader PCC literature [2, 3, 29]. A critical observation was that some symptoms, such as loss of smell/taste (21.4%; n = 9/42) and unusual chest pain (35.7%; n = 15/42), were reported exclusively by CYP continuing to meet the PCC definition 3.5-years post-infection (although not statistically different from those no longer meeting the PCC definition 3.5-years post-infection). Notably, these symptoms also featured prominently in our qualitative analysis. This overlap may suggest the existence of symptom constellations specific to persistent PCC, which could have implications for future monitoring and diagnosis. We identified notable mental health difficulties in the qualitative data. CYP described anxiety, distress, and frustration linked to ongoing symptoms, and resulting consequences to education and activities. Quantitatively, there was no evidence of difference between those who continued to meet the PCC definition vs those who did not at 3.5 years post-infection. Several possibilities could explain these findings. For example, the validated scales used may not be sufficiently sensitive to capture PCC specific distress (e.g., where difficulties relate to fluctuating symptoms or uncertainty). In addition, the small sample size may have limited statistical power to detect meaningful differences. Alternatively, these findings may reflect the broader context, with survey data showing that around 20% of CYP in England had a probable mental disorder in 2023 [30], highlighting the generally poor state of mental health in this population [31].

## Conclusions

We sought to provide new insights into long-term impact and outcomes of persistent PCC in CYP drawing on both qualitative and quantitative descriptions of the condition from CYP 3.5-years post-infection. While most PCC studies have relied on quantitative approaches [2, 11, 32], relatively few have incorporated qualitative methods to explore the breadth and impact of PCC symptoms [26, 33]. Even fewer integrate both methodologies to study PCC [34]. We view this as a methodological gap and opportunity to harness the strengths of both approaches in addressing ongoing challenges posed by PCC in CYP. We found that 84% of CYP who persistently meet the PCC definition at 24-months post-infection, continued to meet the PCC definition 3.5-years post-infection. CYP who continued to meet the PCC definition 3.5-years post-infection had high symptom burden and healthcare engagement. Quantitative and qualitative findings converged to demonstrate that tiredness was a common and central symptom of PCC. CYP experiencing PCC up to 3.5-years post-infection also present with multiple symptoms with wide-ranging severity that impact daily life and self-rated health. Current guidance on treating PCC recommends multidisciplinary rehabilitation and symptom-based approaches [35]. Ongoing trials, service developments (integrating physical and mental health [36]) and service evaluations are crucial to establish best practice for managing persisting PCC in CYP.

## Supplementary Information

Supplementary Material 1.

## Data Availability

Data from the Children and Young People with Long COVID (CLoCk) study are publicly available via the UK Data Service (ID: 9203); DOI (https://doi.org/10.5255/UKDA-SN-9203-1). The 3.5-year questionnaire used in this study was adapted from questionnaires previously published in Nugawela MD, Pinto Pereira SM, Rojas NK, McOwat K, Simmons R, Dalrymple E, et al. Data Resource Profile: the Children and Young People with Long COVID (CLoCk) Study. *Int J Epidemiol*. 2023; dyad158. 10.1093/ije/dyad158 (reference 12). The full version of the questionnaire is provided in the supplementary materials. Permission to use the Strengths and Difficulties Questionnaire was obtained from the instrument’s copyright holders prior to study commencement.

## Abbreviations

PCC: Post-COVID-19 Condition
CYP: Children and Young People
CLoCk: Children and Young People with Long COVID (study name)
PCR: Polymerase Chain Reaction (test)
IMD: Index of Multiple Deprivation
SDQ: Strengths and Difficulties Questionnaire
SWEMWBS: Short Warwick Edinburgh Mental Wellbeing Scale
