# Supplementary material for: Exploring post-Covid-19 condition in children and young people 3.5 years after infection: a mixed-methods analysis from the CLoCk study
Source: BMC Public Health. 2026 May 19;26:2136. doi: 10.1186/s12889-026-27520-z (PMC13359411; doi:10.1186/s12889-026-27520-z)
Supplement: Supplementary file 1 — Supplementary Material 1. [file 12889_2026_27520_MOESM1_ESM.docx]

**Supplementary Information**

| **Supplementary table 1:**Differences 3.5-years post index-infection for the quantitative analytical sample comparing those meeting vs not meeting the PCC definition 3.5-years post-infection (Mean differences in validated scales or differences in proportions (95% confidence intervals)) | |
| --- | --- |
|  | **Mean Difference** |
| **Strengths and Difficulties Questionnaire (SDQ)** |  |
| Total Difficulties | -1.06 (-5.57, 3.45) |
| Emotional symptoms | -0.93 (-2.68, 0.81) |
| Conduct problems | 0.55 (-0.80, 1.90) |
| Hyperactivity/inattention | 0.16 (-1.63, 1.95) |
| Peer relationship problem | -0.84 (-2.43, 0.76) |
| **Short Warwick-Edinburgh Mental Wellbeing Scale (SWEMWBS)** | 0.34 (-2.80, 3.50) |
|  | **Difference in Proportions** |
| **Chalder fatigue scale (CFS)** |  |
| Difference in proportion meeting case-ness | 5.3% (-18.8, 29.6) |
|  |  |
| **Specific symptoms** |  |
| Fever | 7.1% (-0.6, 14.9) |
| Chills | 42.9% (27.9, 57.8) |
| Persistent cough | 42.9% (27.9, 57.8) |
| Tiredness | 25.0% (5.0, 55.0) |
| Shortness of breath | 21.4% (-15.8, 58.7) |
| Loss of smell/taste | 21.4% (9.0, 33.8) |
| Unusually hoarse voice | 4.8% (-1.7, 11.0) |
| Unusual chest pain | 36.7% (21.2, 50.2) |
| Unusual abdominal pain | 26.2% (12.9, 39.5) |
| Diarrhoea | 16.7% (5.4, 27.9) |
| Headaches | 24.4% (-12.2, 61.0) |
| Unusual eye-soreness | 11.3% (-15.0, 37.6) |
| Skipping meals | 20.8% (-6.2, 47.8) |
| Dizziness or light-headedness | 35.1% (7.7, 62.6) |
| Sore throat | -13.1% (-44.7, 18.5) |
| Unusual strong muscle pains | 21.4% (9.0, 33.8) |
| Earache or ringing in ears | 31.0% (17.0, 44.9) |
| Raised welts on skin or swelling | 0.0% |
| Red/purple sores/blisters on feet | 0.0% |
| Sleeping difficulties | 43.5% (7.9, 79.0) |
| Other | 11.9% (2.1, 21.7) |
| Did you/your parent talk to a doctor about your PCC symptoms? | 46.5% (18.8, 74.1) |
| Did you stay overnight in hospital for PCC? | 2.5% (-2.3, 7.3) |

**Supplementary Fig 1: Data flow diagram for the target sample and analytical sample under consideration***

*For a detailed overview of the CLoCk sampling strategy see *Nugawela*et al*. Int J Epidemiol 2024 53(1)*;

# Children & young people with Long Covid (CLoCk) study

Health questionnaire for children and young people to answer directly.

These questions are to be answered by the Young Person themselves. If you need any help, please ask a parent, relative, carer or friend to help you.

For questions that ask for a particular date, don’t worry if you can’t remember it exactly, just enter the closest date.

The questions **do not** need to be completed in one go but can be paused and continued at a later time - just remember to click save.

All of the information which you provide will be kept confidential and no information that could be used to identify you will be shared outside the research team studying Long Covid in young people.

Please enter the unique personal number which we sent you asking to take part:

# About you (if unsure, best estimate)

Please complete the survey below.

Thank you!

How old are you? (years) How tall are you? (cm)

What is your weight now? (kg)

# Long Covid and you

## How many times have you had/do you think you have had covid?

- 0
- 1
- 2
- 3+
- Unsure

## How often did you do a test each time you thought you had Covid?

- Yes, always
- Yes, some of the time
- Never
- I can’t remember

**The following questions are about Long Covid.**

**Although most people recover from Covid-19, a sub group continue to experience persistent symptoms after infection with SARS-CoV-2 virus. This problem has been termed ‘Long Covid’**

## Did you/your parents talk to the doctor about your Long Covid symptoms?

- Yes, always
- Yes, some of the time
- Never
- I can’t remember

## Did you have to stay overnight in hospital for Long Covid?

- Yes, always
- Yes, some of the time
- Never
- I can’t remember

## How many COVID-19 vaccines have you had?

- 0
- 1
- 2
- 3
- 4+
- I can’t remember

# About your health at the moment

How much do you agree with the following statement?

## "I have fully recovered from COVID-19"

- 0 - Strongly Disagree
- 1
- 2
- 3
- 4
- 5 - Neutral
- 6
- 7
- 8
- 9
- 10 - Strongly Agree

## How do you feel right now?

- I feel as healthy as normal
- I am not feeling quite right

## Do you have a fever?

- Yes
- No

## Do you feel chills or shivers (feel too cold)?

- Yes
- No

## Do you have a persistent cough (coughing a lot for more than an hour, or 3 or more coughing episodes in 24 hours)?

- Yes
- No

## Are you experiencing unusual fatigue/tiredness?

- No
- Mild fatigue
- Severe fatigue - I struggle to get out of bed

## Are you experiencing problems with your sleep, including getting to sleep, waking in the night or waking early?

- Yes
- No

## If yes, please describe

Are you experiencing unusual shortness of breath?

- No
- Yes, mild symptoms - slight shortness of breath during ordinary activity
- Yes, significant symptoms - breathing is comfortable only at rest
- Yes, severe symptoms - breathing is difficult even at rest

## What are your current symptoms? (Please tick all that apply)

- loss of smell/taste
- unusually hoarse voice
- unusual chest pain or tightness in your chest
- unusual abdominal pain
- diarrhoea
- headache
- unusual eye-soreness or discomfort (e.g. light sensitivity, excessive tears, or pink/red eye)
- skipping meals
- dizziness or light-headedness
- sore throat
- unusual strong muscle pains
- earache or ringing in your ears (tinnitus)
- raised, red, itchy welts on the skin or sudden swelling of the face or lips
- red/purple sores or blisters on your feet, including your toes
- other

## Are there any other important symptoms you want to share with us?

Since the start of your symptoms, have you had a period longer than one week with none of the above symptoms at all (where you were back to how you were pre-Covid)

- Yes (I have had a period of one week or more since a positive COVID test with none of the above symptoms)
- No (My symptoms have been continuous since they started)
- Not applicable

How severe would you rate your symptoms? 0 (not severe at all) to 100 (extremely severe)

How much do your symptoms affect your functioning? 0 (not at all) to 100 (extremely)

Please describe here how severe your symptoms have been and how they have affected your daily life

# How you feel about your overall health

Describing your health ***TODAY***

Under each heading, please tick the ONE box that describes **your health TODAY**

ED-5D-Y Questionnaire UCLA Loneliness Scale

We would like to know how good or bad your health is **TODAY**

This scale is numbered from 0 to 100%

**100% means the best health** you can think of

**0% means the worst health** you can think of.

Please look at the scale and select the number for your health TODAY

## Today

- 0
- 5
- 10
- 15
- 20
- 25
- 30
- 35
- 40
- 45
- 50
- 55
- 60
- 65
- 70
- 75
- 80
- 85
- 90
- 95
- 100

# Long Covid and your family

Has Long Covid affected your family members (i.e., mother, father, brother, sister, grandparent etc)?

## In your house

Yes No Don't know

Has anyone been to hospital with Long Covid in your household?

❑ ❑ ❑

If yes, who?

Has anyone been in intensive care (ICU) with Long Covid in your household?

❑ ❑ ❑

If yes, who?

Has anyone died from Long Covid in your household?

❑ ❑ ❑

If yes, who?

Does anyone have ongoing Long Covid problems in your household?

❑ ❑ ❑

If yes, who?

## Use this space to explain how Long Covid has affected people in your household:

In your extended family (Grandparents, aunts, uncles etc)

Yes No Don't know

Has anyone been to hospital with Long Covid in your extended family?

❑ ❑ ❑

If yes, who?

Has anyone been in intensive care (ICU) with Long Covid in your extended family?

❑ ❑ ❑

If yes, who?

Has anyone died from Long Covid in your extended family?

❑ ❑ ❑

If yes, who?

Does anyone have ongoing Long Covid problems in your extended family?

❑ ❑ ❑

If yes, who?

Use this space to explain how Long Covid has affected people in your extended family:

**Wellbeing** Strengths and Difficulties (SDQ) Questionnaire Chalder Fatigue Scale The Short Warwick-Edinburgh Mental wellbeing scale (SWEMWBS)

# Long Covid and treatment

## Are you currently **awaiting** treatment for Long COVID?

- Yes
- No
- Not applicable

## Are you currently **receiving** treatment for Long COVID?

- Yes
- No
- Not applicable

Use this space to describe what kind of treatment are you receiving for Long COVID and if it is helping with your symptoms:

Are you currently **receiving** treatment for any other condition?

- Yes
- No
- Not applicable

## Are you currently **awaiting** treatment for any other condition?

- Yes
- No
- Not applicable

Use this space to describe your condition, what kind of treatment are you receiving, and if it is helping with your symptoms:

Please use this space if there is anything else you would like to tell us about Long COVID and how it has affected you. Please consider its impacts on your health, wellbeing, education and friendships in your response.

Please use this space if there is anything else you would like to tell us about your health or how the pandemic or lockdown have affected you.

This research study cannot offer treatment. If you feel you would like some help, please contact

- your GP
- ChildLine [www.childline.org.uk](http://www.childline.org.uk/)
- NHS 111 111.nhs.uk/, or call on 111
- NHS Long Covid information [www.england.nhs.uk/coronavirus/post-covid-syndrome-long-covid/](http://www.england.nhs.uk/coronavirus/post-covid-syndrome-long-covid/)
- Shout giveusashout.org/, or text 85258
- Long Covid Kids [www.longcovidkids.org](http://www.longcovidkids.org/)

This research study cannot offer treatment. If you feel you would like some help, please contact

- your GP
- ChildLine [www.childline.org.uk](http://www.childline.org.uk/)
- NHS 111 111.nhs.uk/, or call on 111
- NHS Long Covid information [www.england.nhs.uk/coronavirus/post-covid-syndrome-long-covid/](http://www.england.nhs.uk/coronavirus/post-covid-syndrome-long-covid/)
- Shout giveusashout.org/, or text 85258
- Long Covid Kids [www.longcovidkids.org](http://www.longcovidkids.org/)
